# Elevated late-life blood pressure may maintain brain oxygenation and slow amyloid-β accumulation at the expense of cerebral vascular damage

**DOI:** 10.1093/braincomms/fcad112

**Published:** 2023-04-04

**Authors:** Hannah M Tayler, Robert MacLachlan, Özge Güzel, J Scott Miners, Seth Love

**Affiliations:** Dementia Research Group, Institute of Clinical Neurosciences, Bristol Medical School, University of Bristol, Bristol, BS10 5NB, UK; Dementia Research Group, Institute of Clinical Neurosciences, Bristol Medical School, University of Bristol, Bristol, BS10 5NB, UK; Dementia Research Group, Institute of Clinical Neurosciences, Bristol Medical School, University of Bristol, Bristol, BS10 5NB, UK; Dementia Research Group, Institute of Clinical Neurosciences, Bristol Medical School, University of Bristol, Bristol, BS10 5NB, UK; Dementia Research Group, Institute of Clinical Neurosciences, Bristol Medical School, University of Bristol, Bristol, BS10 5NB, UK

**Keywords:** hypertension, Alzheimer’s disease, vascular dementia, cerebral ischaemia, blood–brain barrier

## Abstract

Hypertension in midlife contributes to cognitive decline and is a modifiable risk factor for dementia. The relationship between late-life hypertension and dementia is less clear. We have investigated the relationship of blood pressure and hypertensive status during late life (after 65 years) to post-mortem markers of Alzheimer’s disease (amyloid-β and tau loads); arteriolosclerosis and cerebral amyloid angiopathy; and to biochemical measures of ante-mortem cerebral oxygenation (the myelin-associated glycoprotein:proteolipid protein-1 ratio, which is reduced in chronically hypoperfused brain tissue, and the level of vascular endothelial growth factor-A, which is upregulated by tissue hypoxia); blood–brain barrier damage (indicated by an increase in parenchymal fibrinogen); and pericyte content (platelet-derived growth factor receptor β, which declines with pericyte loss), in Alzheimer’s disease (*n* = 75), vascular (*n* = 20) and mixed dementia (*n* = 31) cohorts. Systolic and diastolic blood pressure measurements were obtained retrospectively from clinical records. Non-amyloid small vessel disease and cerebral amyloid angiopathy were scored semiquantitatively. Amyloid-β and tau loads were assessed by field fraction measurement in immunolabelled sections of frontal and parietal lobes. Homogenates of frozen tissue from the contralateral frontal and parietal lobes (cortex and white matter) were used to measure markers of vascular function by enzyme-linked immunosorbent assay. Diastolic (but not systolic) blood pressure was associated with the preservation of cerebral oxygenation, correlating positively with the ratio of myelin-associated glycoprotein to proteolipid protein-1 and negatively with vascular endothelial growth factor-A in both the frontal and parietal cortices. Diastolic blood pressure correlated negatively with parenchymal amyloid-β in the parietal cortex. In dementia cases, elevated late-life diastolic blood pressure was associated with more severe arteriolosclerosis and cerebral amyloid angiopathy, and diastolic blood pressure correlated positively with parenchymal fibrinogen, indicating blood–brain barrier breakdown in both regions of the cortex. Systolic blood pressure was related to lower platelet-derived growth factor receptor β in controls in the frontal cortex and in dementia cases in the superficial white matter. We found no association between blood pressure and tau. Our findings demonstrate a complex relationship between late-life blood pressure, disease pathology and vascular function in dementia. We suggest that hypertension helps to reduce cerebral ischaemia (and may slow amyloid-β accumulation) in the face of increasing cerebral vascular resistance, but exacerbates vascular pathology.

## Teaser

Tayler *et al*. report a complex relationship between high blood pressure in late life (≥65 years), vascular function and Alzheimer’s disease pathology. High blood pressure in late life may help to maintain brain perfusion and oxygenation and reduce amyloid-β plaque at the expense of increased arteriolosclerosis and blood–brain barrier damage.

## Graphical Abstract

**Graphical Abstract:**
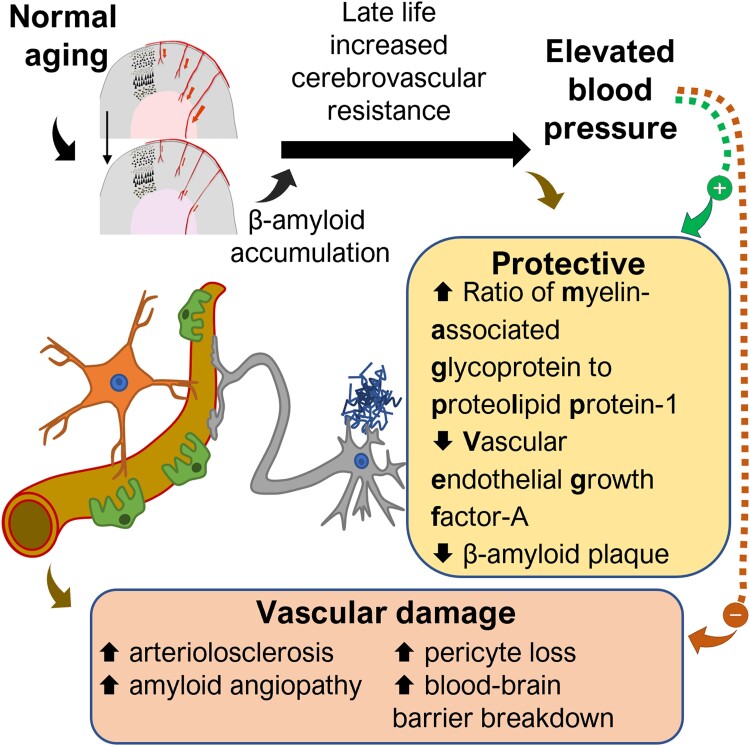

See Magaki and Vinters (https://doi.org/10.1093/braincomms/fcad127) for a scientific commentary on this article.

## Introduction

Hypertension in midlife contributes to cognitive decline and is a modifiable risk factor for dementia. The Honolulu-Asia study found a robust association between midlife hypertension (≥160/95 mmHg) at 45–68 years and clinical diagnosis of vascular dementia and Alzheimer’s disease in late life.^1^ The relative risk for dementia after midlife hypertension is 1.6 [95% confidence interval (CI) 1.2–2.2].^2^ Targeting raised blood pressure (BP) in midlife may protect against later cognitive decline and dementia, including Alzheimer’s disease.^3–5^

Hypertension is also a strong risk factor for stroke, which is itself responsible for cognitive impairment and dementia in 15–70% of survivors.^6,7^ Sustained hypertension in midlife is associated with increased small vessel disease, white matter (WM) hyperintensities,^8^ cerebral microinfarcts^9^ and reduced brain volume.^10–12^ The burden of cerebral small vessel disease increases with the duration of hypertension.^13^ Sustained high BP in midlife causes structural and functional cerebral vascular changes that, over time, increase the risk of inadequate perfusion of the brain, cognitive decline and dementia (reviewed^14^). Blood–brain barrier (BBB) leakiness, endothelial injury and impaired neurovascular coupling, due in part to pericyte damage, are probable early contributors to the onset of small vessel disease and cognitive decline.^15^

Elevated midlife BP may also be associated with increased amyloid-β (Aβ) and tau pathology in Alzheimer’s disease. In the Honolulu-Asia aging study, elevated systolic BP (SBP) (≥160 mmHg) was associated with increased cortical and hippocampal neuritic plaque load and elevated diastolic BP (DBP) (≥95 mmHg) with an increase in neurofibrillary tangles.^1^ Similar findings were reported in other studies.^16,17^ Elevated midlife DBP was associated with higher plasma Aβ in the Honolulu-Asia study.^18^ Another study found a U-shaped relationship between midlife SBP (but not DBP) and plaque density.^19^ We previously reported that Aβ plaque load was increased in hypertensive subjects.^20^ The Systolic Blood Pressure Intervention Trial - Memory and Cognition IN Decreased Hypertension (SPRINT MIND) clinical trial (*n* = 9361, mean age 67) found that intensive versus standard control of BP (SBP < 120 versus < 140 mmHg) did not significantly lower the rate of probable dementia but was associated with a reduced risk of mild cognitive impairment.^21^ However, that trial covered a wide age range, from 50 years upwards (28.2% of participants were ≥75 years).

The relationship of late-life hypertension to dementia and its associated pathologies is less clear-cut. Arvanitakis *et al*.^22^ found an association between mean late-life SBP (but not DBP) and tangle but not plaque pathology. In some studies, late-life hypertension did not influence the risk of cognitive decline and Alzheimer’s disease^5,23–25^; in others, higher SBP, lower DBP and higher BP variability were all associated with higher dementia risk.^26–29^ Verghese *et al.*^30^ reported that elevated BP was protective against dementia in those over 70. Several large longitudinal studies have documented a decline in BP in the years immediately preceding dementia. In middle-aged women over 37 years (*n* = 707), SBP declined more steeply over a 5-year period in those who developed dementia than in those who did not.^31^ Steep declines in BP preceding onset of dementia or clinical diagnosis of Alzheimer’s disease were also observed in the Kungsholmen project^32,33^ and in those over 78 years in the Adult Changes in Thought Study (*n* = 2356).^34^ Cheng *et al.*^35^ calculated that the risk of developing dementia was highest when hypertension was ‘stabilized’ and lowest in participants with normal BP who developed hypertension over the 6-year study period. In very late life, hypertension is protective against dementia.^36,37^ Although in several studies the treatment of hypertension in midlife reduced the long-term risk or progression of dementia,^26,38,39^ in frail or elderly patients, particularly if already cognitively impaired, lowering SBP below 130 mmHg was associated with increased mortality.^40^

In the present study, the objectives were to better understand the complex interactions between late-life BP and markers of Alzheimer’s disease and vascular pathology in a large pathological study. We have used a combination of histological and biochemical methods to analyse the relationships between late-life (after 65 years) SBP and DBP, Alzheimer’s disease pathology (Aβ and tau), non-amyloid small vessel disease (arteriolosclerosis) and cerebral amyloid angiopathy, and markers of cerebral vascular function: ante-mortem cerebral oxygenation [vascular endothelial growth factor-A (VEGF-A) and myelin-associated glycoprotein:proteolipid protein-1 (MAG:PLP1)], BBB integrity [fibrinogen (FGA)] and pericyte content [platelet-derived growth factor receptor β (PDGFRB)].

## Materials and methods

### Study cohort

Post-mortem human brain tissue was obtained from the South West Dementia Brain Bank (SWDBB), University of Bristol, UK. The study was approved by the management committee of the SWDBB (Human Tissue Authority licence number 12273) under the terms of Bristol Research Ethics Committee approval (18/SW/0029). The right cerebral hemisphere had previously been fixed in buffered formalin for 3 weeks and was used for pathological assessment. The left cerebral hemisphere had been sliced and frozen at −80°C. The frontal cortex (FC) (Brodmann area 6) and superficial underlying WM and parietal cortex (PC) (Brodmann area 7) and superficial underlying WM were used in this study. Most of the brains had been dissected within 72 h of death (Table 1).

**Table 1:** Summary of cohort data

	Controls		Alzheimer’s disease		Mixed		Vascular dementia	
	*N* = 100		*N* = 75		*N* = 31		*N* = 20	
Age (y)	84 (78–90)		80 (76–85)		88 (84–92)		83.5 (77.5–89)	
Sex (F:M)	47:53		37:38		22:9		10:10	
Post-mortem delay (h)	43 (33–56)		35 (22–53)		36 (24–46)		43 (29–58)	
Age of dementia onset (y)	—		73 (66–80)		79 (73–87)		79 (71–86)	
Duration of dementia (y)	—		7 (3–10)		8 (5–11)		4 (2–6)	
Braak tangle stage								
0-II	82		0		0		15	
III-IV	18		12		11		5	
V-VI	0		63		20		0	
BP readings (*n*)	19 (8–40)		10 (4–16)		23 (15–35)		8 (5–16)	
Late-life DBP mean (mmHg)	78 (73–83)		80 (76–87)		81 (74–85)		80 (78–84)	
Late-life SBP mean (mmHg)	140 (130–149)		142 (130–150)		148 (136–152)		145 (133–157)	
Pre-dementia DBP mean (mmHg)			81 (77–89)		87 (80–91)		85 (83–87)	
			*N* = 32		*N* = 26		*N* = 5	
Pre-dementia SBP mean (mmHg)			143 (127–151)		148 (135–160)		145 (144–150)	
			*N* = 32		*N* = 26		*N* = 5	
Arteriolosclerosis score	Frontal	Parietal	Frontal	Parietal	Frontal	Parietal	Frontal	Parietal
0	18	24	7	11	1	1	0	0
1	41	43	38	26	6	5	1	4
2	30	18	22	21	17	19	9	7
3	5	3	7	5	7	5	10	8
Cerebral amyloid angiopathy score	Frontal	Parietal	Frontal	Parietal	Frontal	Parietal	Frontal	Parietal
0	69	62	19	21	13	14	16	14
1	13	13	22	20	4	6	1	2
2	13	15	21	22	8	4	3	2
3	5	5	13	11	6	7	0	0

Age, post-mortem delay, BP readings and DBP and SBP means are presented as median and IQR. Arteriolosclerosis scores were not available for *n* = 6 controls and *n* = 1 Alzheimer’s disease case (frontal) and *n* = 12 controls, *n* = 12 Alzheimer’s disease, *n* = 1 mixed and *n* = 1 vascular dementia cases (parietal). Cerebral amyloid angiopathy scores were not available for *n* = 5 controls, *n* = 1 Alzheimer’s disease and *n* = 2 vascular dementia cases (parietal only). Mean late-life DBP and SBP were calculated from all available BP measurements after the age of 65 for every subject. Pre-dementia DBP and SBP means were calculated, where possible, from all available BP measurements between the age of 65 and 5 years prior to dementia diagnosis.

Abbreviations: DBP = diastolic BP; mixed = mixed Alzheimer’s/vascular dementia; SBP = systolic BP.

The age-matched control brains (*n* = 100) were from donors with no history of dementia, few or absent neuritic plaques, a Braak tangle stage of III or less and no other neuropathological abnormalities. The Alzheimer’s disease cases (*n* = 75) had a clinical diagnosis of dementia made during life and either intermediate or high Alzheimer’s disease neuropathological change according to the National Institute on Aging - Alzheimer’s Association (NIA-AA) guidelines.^41^ The vascular dementia cases (*n* = 20) had a clinical history of dementia with histopathological evidence of multiple infarcts/ischaemic lesions and moderate to severe atheroma and/or arteriosclerosis and only occasional neuritic plaques. The Alzheimer’s/vascular mixed dementia cases (*n* = 31) had multiple infarcts/ischaemic lesions and moderate to severe atheroma and/or arteriosclerosis but also fulfilled the criteria for Alzheimer’s disease.

Mean late-life DBP and SBP were calculated from all available BP measurements after the age of 65. The average number of BP readings was 18, and the minimum number of BP measurements for inclusion was 2. Mean pre-dementia DBP and SBP were calculated (where possible) from all available BP measurements between the age of 65 and 5 years prior to diagnosis. All brain donations to the SWDBB were considered at the time of case selection (2016) and included if (i) the neuropathological criteria were met; (ii) there were clinical data available from National Health Service (NHS) records obtained post-mortem (i.e. BP readings); and (iii) frozen tissue from the selected brain regions was available. The demographic and clinical features of the cohort are shown in Table 1.

### Scoring severity of cerebral amyloid angiopathy and arteriolosclerosis

Cerebral amyloid angiopathy scores were determined in pan-Aβ (4G8) antibody-labelled paraffin sections of frontal and parietal lobes (as for Aβ parenchymal load assessment). Cerebral amyloid angiopathy scores (0–3) represent the extent of amyloid deposition in the cerebral vessels, from none to severe, as previously reported^42^ and based on the method of Olichney *et al.*^43^

The severity of arteriolosclerosis was scored in haematoxylin- and eosin-stained paraffin sections of frontal and parietal lobes. The scores were based on the level of arteriolar vessel wall thickening and associated luminal narrowing in the subcortical WM, on a 0–3 scale representing none, mild, moderate and severe disease, as previously reported.^42,44^

### Assessment of parenchymal Aβ and tau load by immunohistochemistry

Parenchymal Aβ and phospho-tau loads were determined in formalin-fixed paraffin-embedded sections of FC and PC, labelled by a standard 3,3′-diaminobenzidine (DAB) horseradish peroxidase method in a Ventana BenchMark ULTRA automated immunostainer (Roche Tissue Diagnostics, USA), with either a pan-Aβ 4G8^45^ antibody (1:8000) or an AT8 phospho-tau^46^ antibody (1:500). Field fraction analysis of the area of section immunopositive for the relevant antigen was performed using image analysis software (Image-Pro Plus 7, Media Cybernetics, USA), as previously reported.^42^

### Preparation of homogenates for biochemical assays

Brain tissue samples were diluted 20% w/v in 1% sodium dodecyl sulphate (SDS) lysis buffer [1% w/v SDS, 0.1 M sodium chloride (NaCl), 0.01 M Tris hydrochloride (Tris-HCl) (pH 7.6), 1 µg/ml of aprotinin and 1 µM phenylmethylsulphonyl fluoride (PMSF)] and homogenized with 5–10 silica beads (2.3 mm diameter) in a Precellys homogenizer (2 × 15 s at 6000 rpm). Homogenates were aliquoted and stored at −80°C prior to use. All subsequent biochemical assays were conducted blinded to the clinical and pathological variables.

### Enzyme-linked immunosorbent assay measurement of Aβ40 and Aβ42

Insoluble (guanidine-extracted) tissue homogenates were prepared as previously described.^42,47–50^ Aβ40 and Aβ42 were measured by sandwich enzyme-linked immunosorbent assay (ELISA) (DAB140, DAB142; R&D Systems, USA) according to the manufacturer’s instructions. Assays on cortical brain homogenates were performed at a 1:4000 dilution and on WM homogenates at 1:2500.

### Enzyme-linked immunosorbent assay measurement of myelin-associated glycoprotein and proteolipid protein-1

The concentration of MAG was determined by in-house direct ELISA and the concentration of PLP1 by sandwich ELISA (PLP1, SEA417Hu; Cloud-Clone Corp., USA/China), as previously described.^42,44,47,48,51^

### Enzyme-linked immunosorbent assay measurement of vascular endothelial growth factor-A

The VEGF-A level was measured by sandwich ELISA (Human VEGF DuoSet, DY293B, R&D Systems, Oxford, UK) as in our previous studies.^47–49^

### Enzyme-linked immunosorbent assay measurement of fibrinogen

FGA was measured by sandwich ELISA (EH3057; FineTest, China) according to the manufacturer’s instructions (and as in our previous studies^42,49^). To allow comparison of parenchymal FGA (i.e. to adjust for blood content), we also measured haemoglobin (Hb) levels by colorimetric assay (700540; Cayman Chemicals, USA), in a modified 384-well format, at a 1:10 dilution in triplicate, as previously described.^42^ Sample Hb content was interpolated from the linear standard (0.016–0.4 g/dl). The FGA level was adjusted for Hb in each sample relative to a mean adult blood Hb of 14 g/dl (e.g. if a sample contained 0.14 g/dl of Hb, 1% of the FGA was assumed to be of haematogenous origin), although in practice this had minimal impact on the data.

### Enzyme-linked immunosorbent assay measurement of platelet-derived growth factor receptor β

PDGFRB was measured by sandwich ELISA (Human Total PDGFRB DuoSet DYC385, R&D systems, Oxford, UK), as previously.^49^

### Statistical analysis

Statistical analyses and graphs were produced using either GraphPad Prism (version 9 GraphPad Software LLC, USA) or Stata (version MP 17.0, StataCorp LLC, USA). All data sets were assessed for normality of distribution and, if not normally distributed, were either log-transformed or analysed by a non-parametric test, as appropriate. The statistical tests used are indicated in the text.

## Results

We studied 226 cases: 75 Alzheimer’s disease, 20 vascular dementia, 31 mixed Alzheimer’s disease/vascular dementia and 100 age-matched controls. The age at death differed slightly between the groups (Kruskal–Wallis test with Dunn’s post-test): Alzheimer’s disease cases were younger (*P* = 0.0006) than the controls. Gender distribution was evenly distributed across controls, Alzheimer’s disease and vascular dementia groups. The mixed dementia group included proportionately more females, but the difference was not statistically significant (*χ*^2^ (df 3) = 5.6697, *P* = 0.129). The groups were matched approximately for post-mortem delay. The age of dementia onset and duration of disease are shown in Table 1. The age of onset of dementia was youngest in Alzheimer’s disease cases (Dunn’s: Alzheimer’s disease versus mixed, *P* = 0.0055) and the duration of dementia shortest in vascular dementia cases (Dunn’s: Alzheimer’s disease versus vascular dementia, *P* = 0.0247; mixed versus vascular dementia, *P* = 0.0059).

The Alzheimer’s disease and vascular dementia cases had fewer recorded BP measurements; this may reflect the shorter duration of vascular dementia and the lower age of death of Alzheimer’s disease cases than controls.

Mean SBP and DBP and interquartile range (IQR) values for each group are presented in Table 1. DBP was higher in dementia cases than controls in an age- and sex-adjusted model (linear regression: *F* (3, 222) = 4.97, *P* = 0.0023 with *R^2^* of 0.0529) (Supplementary Table 2: model 1). DBP was also higher in the Alzheimer’s disease and vascular dementia subtypes (*F* (5, 220) = 3.27, *P* = 0.0072; Supplementary Table 2: model 2). As BP may fall after dementia diagnosis, we also looked at the BP data for individuals to compare pre-dementia late-life DBP and SBP (i.e. BP measurements from after the age of 65 years but at least 5 years prior to diagnosis) to late-life BP in controls. The data available for this secondary analysis were limited because BP measurements were not always recorded between the age of 65 and dementia diagnosis, and in some cases, dementia commenced before or at 70 years. However, pre-dementia measurements of both DBP and SBP were higher in dementia cases overall (*P* < 0.001 and *P* = 0.021, respectively; *n* = 137; Supplementary Table 3: model 1), and DBP separately in Alzheimer’s disease (*P* = 0.047), mixed dementia (*P* < 0.001) and vascular dementia cases (*P* = 0.013) and SBP was also higher in vascular dementia cases (*P* = 0.037), although it should be noted that the small size of the ‘pure’ vascular dementia cohort limits the power of this last subgroup analysis (Supplementary Table 3: model 2).

### Elevated late-life blood pressure was associated with more severe arteriolosclerosis and cerebral amyloid angiopathy scores

Donors with dementia and elevated late-life DBP had higher frontal and parietal arteriolosclerosis scores. The effect of higher DBP was significant in an ordered logistic regression model, adjusting for the effects of age and dementia subtype (adjusted OR per additional frontal arteriolosclerosis score point 1.07, 95% CI 1.03–1.11, *P* = 0.001; parietal arteriolosclerosis score point 1.05, 95% CI 1.02–1.09, *P* = 0.007; Table 2 and Supplementary Fig. 1). Similarly, donors with elevated late-life SBP had higher frontal and parietal arteriolosclerosis scores (adjusted OR per additional frontal arteriolosclerosis score point 1.03, 95% CI 1.01–1.05, *P* = 0.001; parietal arteriolosclerosis score point 1.02, 95% CI 1.01–1.04, *P* = 0.010; Supplementary Fig. 1 and Supplementary Table 4).

**Table 2:** Ordered logistic regression for arteriolosclerosis score in relation to DBP by dementia subtype

Variables		Unadjusted analysis			Adjusted analysis		
		Odds ratio	95% CI	*P-*value	Odds ratio	95% CI	*P-*value
Frontal arteriolosclerosis score							
Cohort	Alzheimer’s disease	1.38	0.78–2.44	0.275	1.31	0.71–2.40	0.382
	Mixed	5.69	2.62–12.4	**<0**.**001**	5.41	2.52–11.6	**<0**.**001**
	Vascular dementia	19.1	7.53–48.2	**<0**.**001**	17.0	6.39–45.5	**<0**.**001**
Age (**y**)		1.03	1.00–1.06	0.074	1.04	1.00–1.07	**0.030**
DBP (after 65 years)		1.07	1.04–1.10	**<0.001**	1.07	1.03–1.11	**0.001**
Parietal arteriolosclerosis score							
Cohort	Alzheimer’s disease	2.09	1.11–3.94	**0**.**022**	2.28	1.18–4.40	**0**.**014**
	Mixed	8.79	4.19–18.4	**<0**.**001**	8.19	3.91–17.2	**<0**.**001**
	Vascular dementia	18.1	5.74–57.3	**<0**.**001**	17.0	4.63–62.3	**<0**.**001**
Age (**y**)		1.04	1.01–1.07	**0.022**	1.05	1.01–1.08	**0.005**
DBP (after 65 years)		1.06	1.03–1.09	**<0.001**	1.05	1.01–1.09	**0.007**

Frontal *n* = 219, parietal *n* = 200.

Abbreviations: CI = confidence interval; DBP = diastolic blood pressure; y = years.

The unadjusted ordered logistic regression analysis shows the effects of each of the separate variables (DBP, disease subtype, age) on frontal arteriolosclerosis score. The adjusted analysis is the same regression of DBP on frontal arteriolosclerosis score in the presence of the covariates, age and disease subtype. Significant *P*-values are denoted in **bold**.

SBP was associated with frontal cerebral amyloid angiopathy severity in an ordered logistic regression model, adjusting for the effects of age and dementia subtype (adjusted OR per additional frontal cerebral amyloid angiopathy score point 1.02, 95% CI 1.00–1.03, *P* = 0.038; Supplementary Table 5), but not parietal cerebral amyloid angiopathy severity. DBP was not significantly associated with cerebral amyloid angiopathy severity (not shown).

### Elevated late-life diastolic blood pressure was associated with lower parenchymal Aβ load

**Figure 1:**
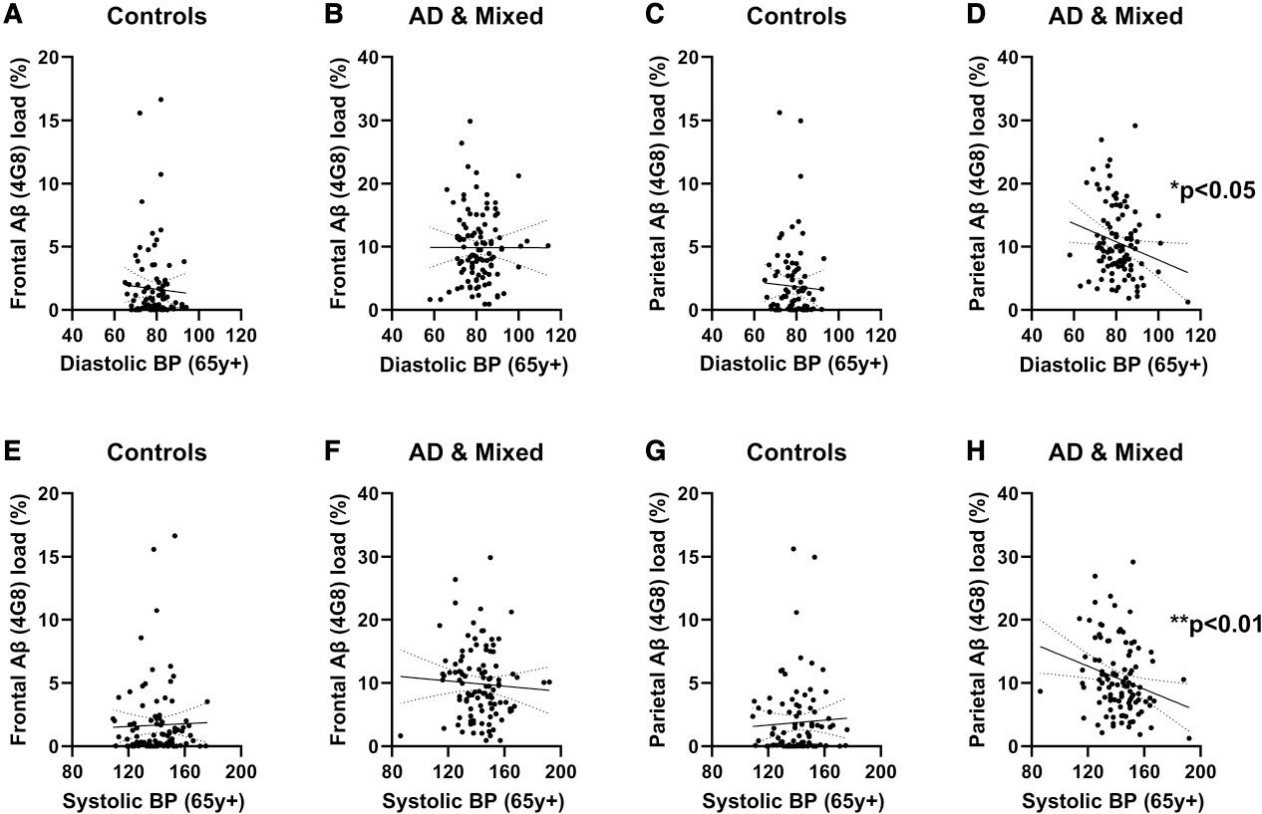
**Elevated DBP and SBP in late life were associated with reduced parenchymal Aβ load in the PC in Alzheimer's disease and mixed dementia.** No relationship was seen between DBP and frontal Aβ load in (**A**) controls (Spearman’s correlation, *n* = 97, *r* = −0.0043, ns) or in (**B**) Alzheimer’s disease and mixed cases (*n* = 106, Spearman’s *r* = −0.0398, ns). No relationship was seen between DBP and parietal Aβ load in (**C**) controls (*n* = 92, *r* = −0.02337, ns). However, late-life DBP was associated with lower parietal Aβ (4G8) load in (**D**) Alzheimer’s disease and mixed dementia cases (*n* = 104, *r* = −0.2050, *P* = 0.0368). Late-life SBP did not correlate with frontal Aβ load in either (**E**) controls (*n* = 97, *r* = 0.05707, ns) or (**F**) Alzheimer’s and mixed dementia cases (*n* = 106, *r* = −0.1189, ns). Late-life SBP also did not correlate with parietal Aβ load in (**G**) controls (*n* = 92, *r* = 0.0907, ns). However, in Alzheimer’s disease and mixed dementia cases, late-life SBP correlated with lower (**H**) parietal Aβ load (*n* = 104, *r* = − 0.2570, *P* = 0.0084). Each point represents a single case. The continuous and interrupted lines indicate the best-fit linear regression and 95% CIs. Abbreviations: Ad = Alzheimer’s disease; BP = blood pressure; FC = frontal cortex; PC = parietal cortex; y = years.

In Alzheimer’s disease and mixed dementia, late-life DBP correlated negatively with parenchymal Aβ load in the PC (*r* = −0.2050, *P* = 0.0368; Fig. 1D) as did late-life SBP (*r* = −0.2570, *P* = 0.0084; Fig. 1H), but these relationships were not significant in controls (Fig. 1C and G) or in either group in the FC (Fig. 1A, B, D and E). We did not find associations between late-life BP and levels of insoluble Aβ40 or Aβ42 in tissue homogenates, or tau load in paraffin sections, in either brain region (data not shown).

### Elevated late-life diastolic blood pressure was associated with less biochemical evidence of reduced oxygenation

**Figure 2:**
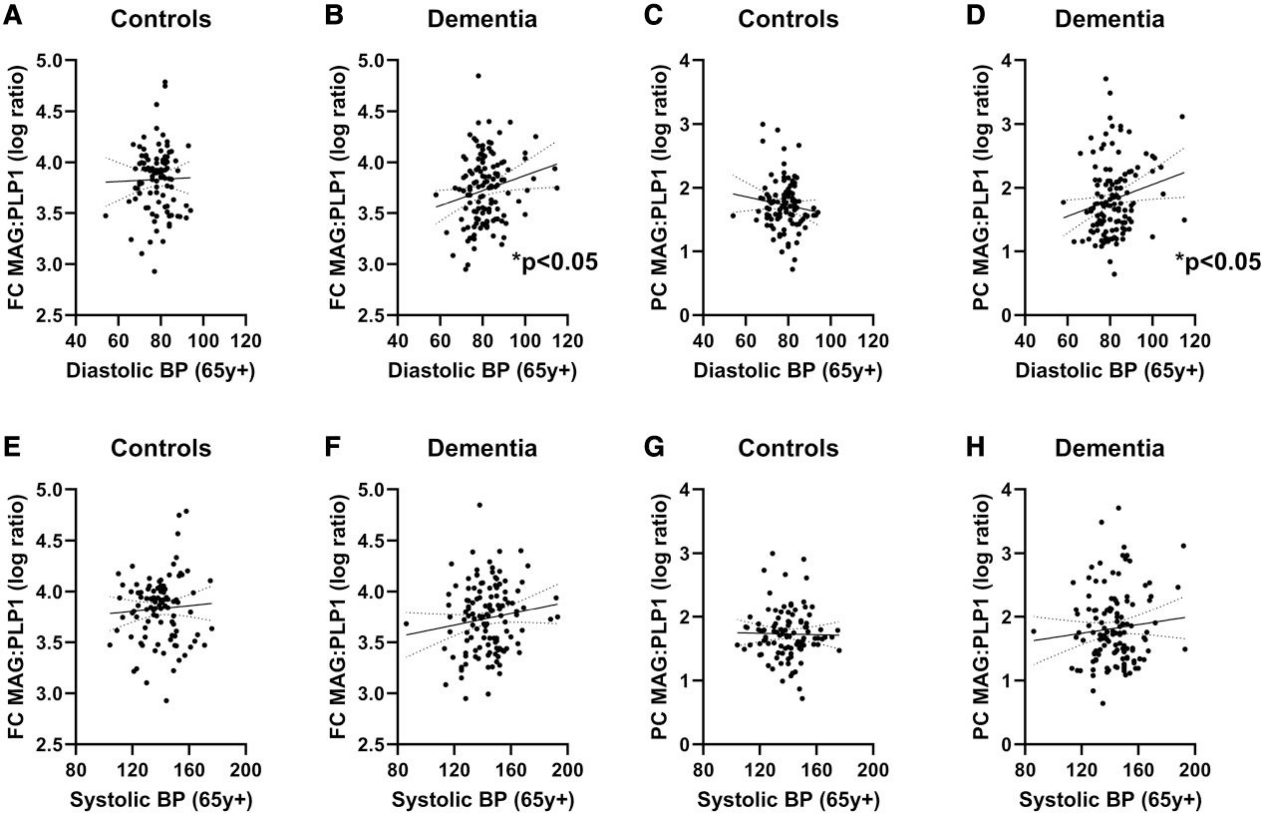
**Elevated DBP in late life was associated with higher cortical MAG:PLP1 in dementia.** Late-life DBP was not significantly associated with FC MAG:PLP1 in (**A**) controls (Pearson’s correlation, ns, *n* = 99) but was associated with higher (**B**) FC MAG:PLP1 (Pearson’s *r* = 0.1985, *P* = 0.0259, *n* = 126) in dementia cases. Similarly, late-life DBP was not associated with (**C**) PC MAG:PLP1 ratios in controls (ns, *n* = 99) but correlated positively with (**D**) PC MAG:PLP1 (*r* = 0.1971, *P* = 0.0282, *n* = 124) ratios in dementia cases. Late-life SBP did not correlate with MAG:PLP1 in dementia cases or controls in either brain region (**E–H**; ns). Each point represents a single case. The continuous and interrupted lines indicate the best-fit linear regression and 95% CIs. Abbreviations: BP = blood pressure; FC = frontal cortex; MAG:PLP1 = myelin-associated glycoprotein:proteolipid protein-1; PC = parietal cortex; y = years.

In all dementia cases (i.e. combining Alzheimer’s disease, mixed and vascular dementia) but not controls, MAG:PLP1 in the FC correlated positively with DBP (Pearson’s *r* = 0.1985, *P* = 0.0259, *n* = 126) and PC (*r* = 0.1971, *P* = 0.0282, *n* = 124) but not with SBP (Fig. 2). MAG:PLP1 in frontal WM or parietal WM was not related to DBP or SBP (Supplementary Fig. 2).

**Figure 3:**
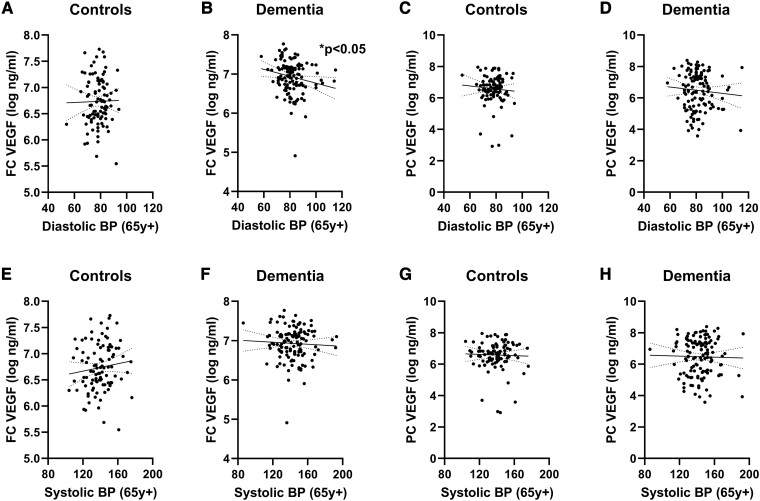
**Elevated DBP in late life was associated with lower VEGF in the FC in dementia.** No relationship was seen between DBP and (**A**) FC VEGF in controls (Pearson’s correlation, ns, *n* = 99). Late-life DBP was associated with lower (**B**) FC VEGF (Pearson’s *r* = −0.1909, *P* = 0.0322, *n* = 126) in dementia cases. No relationship was seen between DBP and PC VEGF in (**C**) controls (ns, *n* = 97) or in (**D**) dementia cases (ns, *n* = 125). Late-life SBP did not correlate with VEGF in controls or dementia cases in either brain region (**E–H**; ns). Each point represents a single case. The continuous and interrupted lines indicate the best-fit linear regression and 95% CIs. Abbreviations: BP = blood pressure; FC = frontal cortex; PC = parietal cortex; VEGF = vascular endothelial growth factor; y = years.

In the FC (Pearson’s *r* = −0.1909, *P* = 0.0322; Fig. 3B) but not the PC (ns; Fig. 3D), the VEGF level, which increases in response to recent brain ischaemia, correlated negatively with late-life DBP in dementia cases. The VEGF level did not correlate in either brain region with late-life DBP in controls (both ns; Fig. 3A and C). VEGF levels did not correlate with late-life SBP in controls or dementia cases in either brain region (ns; Fig. 3E–H).

The VEGF level in the frontal WM also correlated negatively with DBP in dementia cases (*r* = −0.2267, *P* = 0.0107; Supplementary Fig. 3) but not controls. Again, the relationships with SBP were not significant. VEGF in the parietal WM did not vary with respect to DBP or SBP in dementia or control cases.

### Elevated late-life diastolic and systolic blood pressure was associated with blood–brain barrier leakiness in dementia

**Figure 4:**
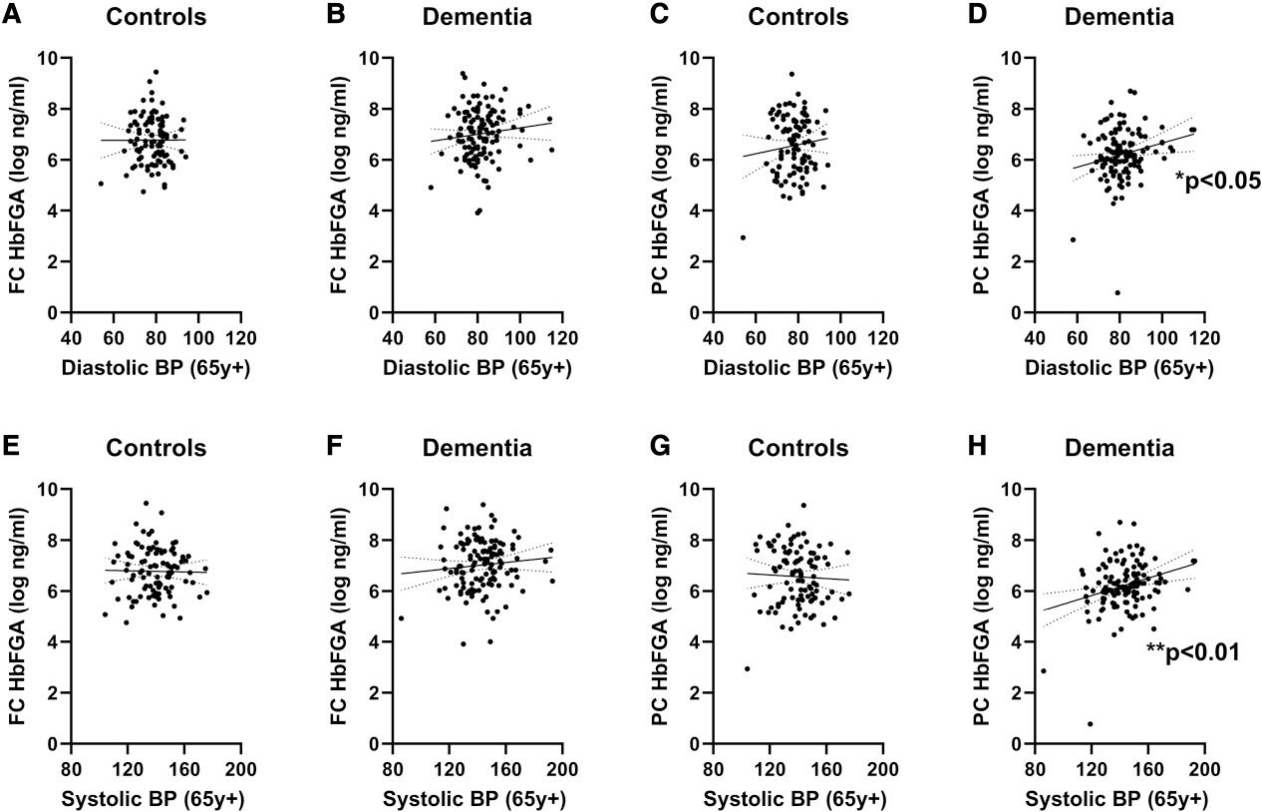
**Elevated DBP and SBP in late life were associated with higher parietal cortical Hb-adjusted FGA levels in dementia.** No relationship was seen between DBP and FC HbFGA in (**A**) controls (Pearson’s correlation, ns, *n* = 99) or in (**B**) dementia cases (ns, *n* = 126). Similarly, no significant relationship was found between DBP and PC HbFGA in (**C**) controls (ns, *n* = 99). Late-life DBP was associated with lower (**D**) PC HbFGA (Pearson’s *r* = 0.2119, *P* = 0.0186, *n* = 123). There was no relationship between late-life SBP and FC HbFGA in either (**E**) controls (ns) or (**F**) dementia cases (ns). There was no relationship between late-life SBP and PC HbFGA in (**G**) controls (ns). However, late-life SBP correlated positively with PC HbFGA in (**H**) dementia cases (Pearson’s *r* = 0.2719, *P* = 0.0023). Each point represents a single case. The continuous and interrupted lines indicate the best-fit linear regression and 95% CIs. Abbreviations: BP = blood pressure; FC = frontal cortex; HbFGA = haemoglobin-adjusted fibrinogen; PC = parietal cortex; y = years.

In the PC, the FGA level (adjusted for Hb content) correlated positively with late-life DBP in the combined dementia cohort (Pearson’s *r* = 0.2119, *P* = 0.0186). This correlation was not seen in the FC in dementia cases (ns) or in controls in either brain region (both ns) (Fig. 4). Late-life SBP also correlated with Hb-adjusted FGA levels in the PC in dementia cases (*r* = 0.2719, *P* = 0.0023) but not controls (ns). There was no correlation in the FC in either dementia cases or controls (both ns).

### Lower late-life systolic blood pressure was associated with pericyte damage in dementia in frontal white matter

**Figure 5:**
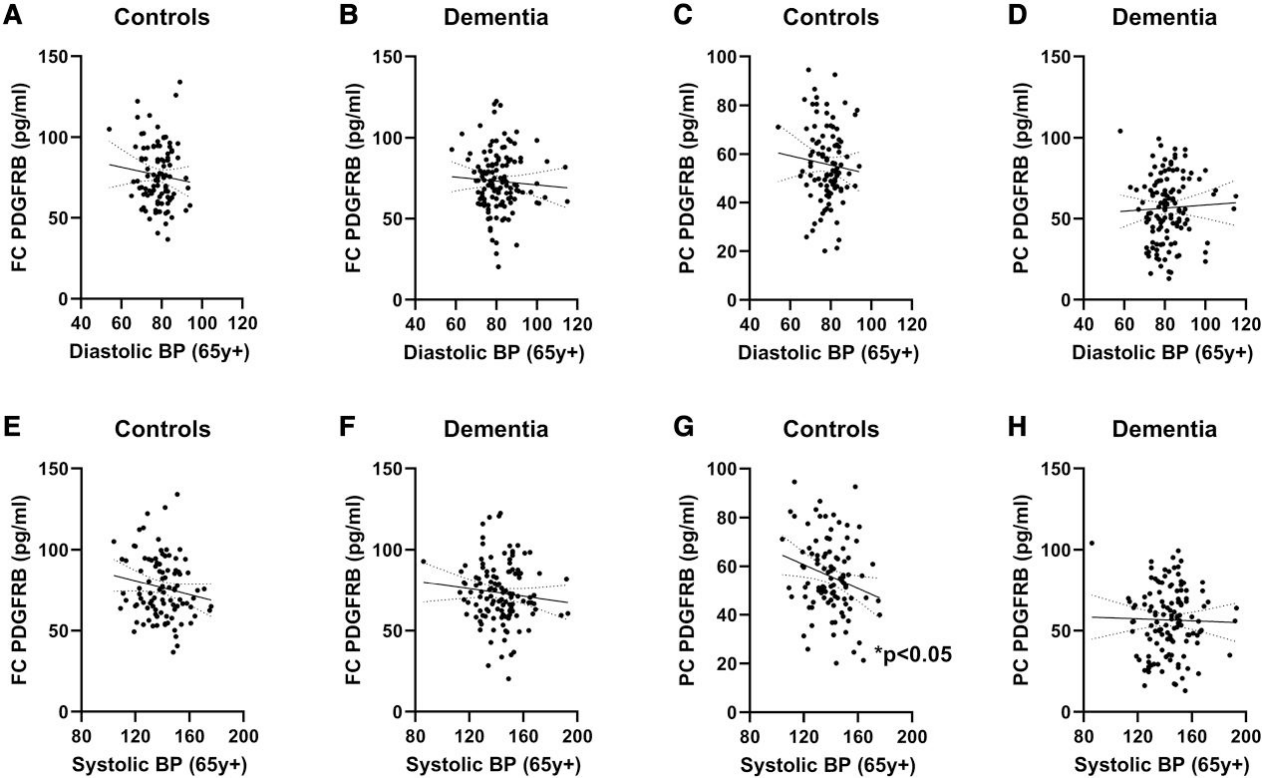
**Elevated SBP in late life was associated with higher PC PDGFRB levels in controls.** Late-life DBP did not correlate (Pearson’s correlation) with (**A**) FC PDGFRB in controls or (**B**) dementia cases or with (**C**) PC PDGFRB in controls or (**D**) dementia cases. There were no significant correlations between late-life SBP and FC PDGFRB in either (**E**) controls or (**F**) dementia cases (both ns). Late-life SBP negatively correlated with PC PDGFRB in (**G**) controls (Pearson’s *r* = −0.2290, *P* = 0.0219, *n* = 100) but not (**H**) dementia cases (ns, *n* = 126). Abbreviations: BP = blood pressure; FC = frontal cortex; PC = parietal cortex; PDGFRB = platelet-derived growth factor receptor β; y = years.

In the FC and PC, PDGFRB did not correlate with DBP in either controls or dementia cases (all ns; Fig. 5A–D). Late-life SBP correlated negatively with PC PDGFRB in controls (*r* = −0.2290, *P* = 0.0219; Fig. 5G) but not dementia cases (ns; Fig. 5H). Late-life SBP did not correlate with FC PDGFRB in either controls or dementia cases (both ns; Fig. 5E and F).

In the frontal and parietal WM, PDGFRB did not correlate with DBP in controls or dementia cases (all ns; Supplementary Fig. 4A–D). Late-life SBP correlated negatively with frontal WM PDGFRB in dementia cases (*r* = −0.1821, *P* = 0.0413; Supplementary Fig. 4F) but not controls (ns; Supplementary Fig. 4E) and not in the parietal WM in either group (both ns; Supplementary Fig. 4G–H).

## Discussion

In this study, we have investigated the relationship between late-life BP and markers of disease and cerebral vascular pathology and dysfunction in people with dementia. We have found late-life BP to be associated with lower parenchymal Aβ load and with biochemical markers of lower ischaemic damage of the cerebral cortex, i.e. may be protective against cerebral hypoperfusion and Aβ accumulation. The relationship between BP and dementia risk is complex. A recent Mendelian randomization study using UK Biobank data found that high BP was associated with reduced late-life risk of developing Alzheimer’s disease.^52^ Our pathological observations support recent studies showing that elevated BP in late life may be protective against Alzheimer’s disease.^26–30^

Our study has weaknesses. The repeated BP measurements were obtained retrospectively from clinical records. Fewer BP measurements were recorded in dementia cases. In addition, changes in the diagnosis and management of hypertension between 1985 and 2019, the period during which the brains for this study were donated, are likely to have impacted the frequency of BP recordings in some cases. A strength of this study is that we have collected all available data from clinical records and used the average of many late-life BP measurements for each individual in our analyses. Generally, in our cohort, BP was monitored more regularly, and hypertension was treated more often for the more recent donations. Some of the regression modelling for this study was limited by the small number of cases in each group, particularly since there were few pure vascular dementia cases, which limited the statistical power of our analyses. We also noted some moderate differences in the average age of the groups. We included age and disease subtype as covariates in our regression modelling partly because of the known associations with arteriolosclerosis and cerebral vascular function.

We found that elevated DBP was associated with more severe arteriolosclerosis in dementia cases. This is in keeping with evidence of a relationship between the severity of arteriolosclerosis and DBP.^22^ Previous studies demonstrated that longer-term exposure to hypertension strengthened the association with cerebral small vessel disease burden.^13^ Higher late-life BP was also associated with an increased number of brain infarcts, and exposure to vascular risk factors, including hypertension, adversely affected WM integrity.^53^ Muller *et al.*^54^ found an association between higher late-life BP and increased risk of WM lesions and cerebral microbleeds in older participants; the effect was most pronounced in those without a history of midlife hypertension.

In the frontal lobe, the severity of cerebral amyloid angiopathy correlated with SBP. Other neuropathological studies did not find SBP to be associated with cerebral amyloid angiopathy severity.^55,56^ However, arterial hypertension is a frequent comorbidity in people with clinically diagnosed cerebral amyloid angiopathy^57^ and has been shown to interact with severe cerebral amyloid angiopathy to increase the risk of infarction.^58^ In Tg2576 mice, Aβ pathology promoted hypertension-induced intracerebral haemorrhage,^59^ and BP-lowering strategies were suggested as a means to reduce the risk of recurrent cerebral amyloid angiopathy-associated intracerebral haemorrhage.^60,61^ However, another study found that hypertensive cerebral amyloid angiopathy patients had lower mortality than normotensive patients.^62^ In hypertensive rats, severe small vessel disease promoted the development of cerebral amyloid angiopathy.^63^

Observational studies have shown that elevated SBP may be protective in older individuals and may be induced as a response to a decline in brain vascular health.^64,65^ Cerebroventricular infusion of Aβ_40_ in rats significantly elevated BP and exacerbated existing hypertension, probably via modulation of autonomic activity.^66^ In cognitively normal elderly individuals, Aβ deposition (measured by florbetapir-Aβ PET) and WM hyperintensity volume both affected rates of neurodegeneration, but these effects were independent with no evidence of interaction.^67^ Both arteriolosclerosis and cerebral amyloid angiopathy lead to luminal narrowing and increased cerebral vascular resistance, which puts the brain at risk of hypoperfusion. An elevation in BP in response to these vascular alterations may be a physiological adaptation that allows the brain to maintain cerebral perfusion (and maintain clearance of Aβ).^68^ The late-life decline in BP that often precedes the onset of dementia may reflect the decompensation of these adaptive mechanisms for maintaining cerebral perfusion (perhaps contributing to Aβ accumulation).

Damage and leakiness of the BBB are associated with cognitive decline in the early stages of Alzheimer’s disease^69^ and vascular mild-cognitive impairment^70^ and underpin the development of small vessel disease.^71^ We recently found that BBB breakdown was elevated in controls and dementia cases with terminal systemic infection^72^ and that BBB damage was more severe in mixed vascular/Alzheimer’s dementia than in pure Alzheimer’s or vascular dementia cases.^42^ In the present study, FGA levels correlated positively with DBP, in keeping with other evidence of a link between hypertension and BBB damage.^73–75^ Dysfunction of the BBB is likely to be but one manifestation of hypertensive vessel wall damage that impairs the clearance of metabolites and impacts Aβ accumulation in Alzheimer’s disease. Damage to pericytes probably contributes to BBB damage in the early stages of Alzheimer’s disease.^69,76^ Here, we show that elevated SBP was inversely related to PDGFRB content in the FC in controls and in the superficial WM in Alzheimer’s disease.

In the present study, we have shown that elevated DBP in individuals aged 65 years and over correlated negatively with parenchymal Aβ load, specifically in the PC. This contrasts with previous reports of a positive relationship between hypertension and elevated parenchymal Aβ and tau; however, participants in those studies had midlife hypertension.^1^ Our findings also suggest that high BP in late life may protect against some other Alzheimer’s disease-associated changes, including chronic cerebral hypoperfusion. This is evidenced by the higher MAG:PLP1 ratio (see Tayler *et al.*,^42^ Barker *et al.*^44, 51^ and Miners *et al.*^48,49^ and reviewed in Love and Miners^77,78^) in brain donors with a history of elevated late-life DBP and the lower levels of VEGF-A.^47^ We previously showed that cerebral hypoperfusion in Alzheimer’s disease is due in part to elevated expression of potent vasoconstrictors, including endothelin-1^48,79^ and angiotensin-II.^80,81^ Aβ peptides themselves have direct and indirect vasoconstrictor actions.^82–85^ In contrast, we found no evidence of a relationship between DBP and tangle pathology. This supports previous data.^22^

## Conclusion

In conclusion, there is a complex relationship between late-life BP and markers of vascular and Alzheimer’s disease pathology in dementia. Elevated late-life DBP is associated with biochemical markers, which indicate lower ischaemia and less accumulation of Aβ, but also with more severe vascular damage, including arteriolosclerosis, cerebral amyloid angiopathy, BBB breakdown and pericyte loss. Late-life elevated BP may be a physiological response to maintain cerebral oxygenation in response to increased cerebral vascular resistance—an adaptation that maintains cerebral blood flow and Aβ homeostasis but, when sustained over a longer period, exacerbates vascular pathology until eventually BP declines, preceding the onset of clinical disease.

## Supplementary Material

fcad112_Supplementary_Data

## Data Availability

The UK Brain Banks Network identifiers of the brains that were used in this study are listed in (Supplementary Table 1). The data that support the findings of this study are available from the corresponding author upon reasonable request.

## Abbreviations

Aβ =: amyloid-β
BBB =: blood–brain barrier
BP =: blood pressure
CI =: confidence interval
DAB =: diaminobenzidine
DBP =: diastolic blood pressure
FC =: frontal cortex
FGA =: fibrinogen
Hb =: haemoglobin
MAG =: myelin-associated glycoprotein
MAG:PLP1 =: myelin-associated glycoprotein:proteolipid protein-1
mixed =: mixed dementia
NaCl =: sodium chloride
NHS =: National Health Service (UK)
NIA-AA =: National Institute on Aging - Alzheimer’s Association
PC =: parietal cortex
PDGFRB =: platelet-derived growth factor receptor β
PMSF =: phenylmethylsulphonyl fluoride
SBP =: systolic blood pressure
SDS =: sodium dodecyl sulphate
SPRINT MIND =: Systolic Blood Pressure Intervention Trial - Memory and Cognition IN Decreased Hypertension
SWDBB =: South West Dementia Brain Bank
Tris-HCl =: Tris hydrochloride
VEGF-A =: vascular endothelial growth factor-A
WM =: white matter
