# Supplementary material for: Elevated late-life blood pressure may maintain brain oxygenation and slow amyloid-β accumulation at the expense of cerebral vascular damage
Source: Brain Commun. 2023 Apr 4;5(2):fcad112. doi: 10.1093/braincomms/fcad112 (PMC10128877; doi:10.1093/braincomms/fcad112)
Supplement: fcad112_Supplementary_Data [file fcad112_supplementary_data.docx]

# **Supplementary tables and figures**

**Supplementary Table 1 of UK Brain Bank Network identifiers**

| BBN_8642 | BBN_8917 | BBN_9323 | BBN_9420 | BBN_24899 |
| --- | --- | --- | --- | --- |
| BBN_8644 | BBN_8927 | BBN_9329 | BBN_9421 | BBN_24902 |
| BBN_8651 | BBN_8944 | BBN_9336 | BBN_9422 | BBN_25025 |
| BBN_8662 | BBN_8952 | BBN_9338 | BBN_9426 | BBN_26009 |
| BBN_8665 | BBN_8960 | BBN_9340 | BBN_9427 | BBN_26011 |
| BBN_8669 | BBN_8964 | BBN_9341 | BBN_9429 | BBN_26012 |
| BBN_8675 | BBN_8969 | BBN_9343 | BBN_9430 | BBN006.26095 |
| BBN_8677 | BBN_8975 | BBN_9344 | BBN_9432 | BBN006.26096 |
| BBN_8684 | BBN_8981 | BBN_9346 | BBN_9433 | BBN006.26348 |
| BBN_8706 | BBN_8983 | BBN_4200 | BBN_9435 | BBN006.28893 |
| BBN_8708 | BBN_8989 | BBN_4205 | BBN_10251 | BBN006.29018 |
| BBN_8717 | BBN_8997 | BBN_9353 | BBN_14398 | BBN006.29102 |
| BBN_8722 | BBN_9016 | BBN_9354 | BBN_14403 | BBN006.29470 |
| BBN_8723 | BBN_9026 | BBN_4208 | BBN_14404 | BBN006.29875 |
| BBN_8728 | BBN_9028 | BBN_4215 | BBN_14406 | BBN006.30034 |
| BBN_8731 | BBN_9031 | BBN_4216 | BBN_19608 | BBN006.30108 |
| BBN_8735 | BBN_9033 | BBN_9359 | BBN_19612 | BBN006.30165 |
| BBN_8739 | BBN_9076 | BBN_4220 | BBN_19613 | BBN006.30186 |
| BBN_8749 | BBN_9078 | BBN_4223 | BBN_19614 | BBN006.30198 |
| BBN_8751 | BBN_9090 | BBN_9361 | BBN_19615 | BBN006.31488 |
| BBN_8756 | BBN_9108 | BBN_4229 | BBN_19616 | BBN006.31497 |
| BBN_8757 | BBN_9110 | BBN_4232 | BBN_19620 | BBN006.31516 |
| BBN_8760 | BBN_9111 | BBN_9365 | BBN_19624 | BBN006.32529 |
| BBN_8770 | BBN_9112 | BBN_9367 | BBN_19626 | BBN006.32544 |
| BBN_8776 | BBN_9122 | BBN_9368 | BBN_19627 | BBN006.32578 |
| BBN_8779 | BBN_9123 | BBN_9369 | BBN_19628 | BBN006.32821 |
| BBN_8787 | BBN_9132 | BBN_9371 | BBN_19630 | BBN006.32845 |
| BBN_8799 | BBN_9134 | BBN_9372 | BBN_22622 | BBN006.34115 |
| BBN_8819 | BBN_9136 | BBN_9377 | BBN_22623 | BBN006.35100 |
| BBN_8829 | BBN_9141 | BBN_9378 | BBN_24337 | BBN006.35414 |
| BBN_8833 | BBN_9154 | BBN_9379 | BBN_22624 |  |
| BBN_8834 | BBN_9164 | BBN_9387 | BBN_22625 |  |
| BBN_8835 | BBN_9173 | BBN_9389 | BBN_24308 |  |
| BBN_8842 | BBN_9186 | BBN_9392 | BBN_24309 |  |
| BBN_8848 | BBN_9192 | BBN_9394 | BBN_24310 |  |
| BBN_8849 | BBN_9198 | BBN_9395 | BBN_24311 |  |
| BBN_8850 | BBN_9200 | BBN_4238 | BBN_24312 |  |
| BBN_8852 | BBN_9221 | BBN_9397 | BBN_24315 |  |
| BBN_8853 | BBN_9224 | BBN_9398 | BBN_24319 |  |
| BBN_8857 | BBN_9261 | BBN_9399 | BBN_24320 |  |
| BBN_8861 | BBN_9263 | BBN_9401 | BBN_24324 |  |
| BBN_8870 | BBN_9274 | BBN_9405 | BBN_24325 |  |
| BBN_8871 | BBN_9293 | BBN_9407 | BBN_24326 |  |
| BBN_8877 | BBN_9296 | BBN_9408 | BBN_24330 |  |
| BBN_8883 | BBN_9299 | BBN_9409 | BBN_24561 |  |
| BBN_8898 | BBN_9303 | BBN_9413 | BBN_24564 |  |
| BBN_8899 | BBN_9311 | BBN_4240 | BBN_24893 |  |
| BBN_8906 | BBN_9313 | BBN_9417 | BBN_24895 |  |
| BBN_8916 | BBN_9315 | BBN_9419 | BBN_24896 |  |

Supplementary Table 2 Elevated late-life DBP in dementia cases

| Variable | | DBP (mmHg) | | | | | | SBP (mmHg) | | | | | |
| --- | --- | --- | --- | --- | --- | --- | --- | --- | --- | --- | --- | --- | --- |
|  | | Unadjusted analysis | | | Adjusted analysis | | | Unadjusted analysis | | | Adjusted analysis | | |
|  | | β | Rob-ust SE | *P*-value | β | Robust SE | *P*-value | β | Robust SE | *P*-value | β | Rob-ust SE | *P*-value |
| Model 1 (dementia vs controls)^a^ | | | | | | | | | | | | | |
| Dementia | | 3.35 | 1.04 | **0.001** | 3.05 | 1.08 | **0.005** | 3.51 | 2.03 | 0.085 | 3.77 | 2.05 | 0.067 |
| Age | | -0.09 | 0.06 | 0.105 | -0.08 | 0.06 | 0.167 | 0.24 | 0.12 | **0.036** | 0.24 | 0.12 | **0.045** |
| Sex: female | | 1.45 | 1.08 | 0.182 | 1.44 | 1.12 | 0.198 | 4.42 | 2.02 | **0.030** | 3.48 | 2.10 | 0.099 |
| Model 2 (dementia subtype vs controls)^b^ | | | | | | | | | | | | | |
| Cohort | AD | 3.62 | 1.21 | **0.003** | 3.35 | 1.26 | **0.008** | 2.39 | 2.28 | 0.297 | 3.34 | 2.32 | 0.151 |
|  | Mixed | 1.08 | 1.49 | 0.471 | 0.83 | 1.49 | 0.580 | 3.71 | 3.09 | 0.231 | 2.21 | 3.06 | 0.471 |
|  | VaD | 5.84 | 2.58 | **0.025** | 5.65 | 2.65 | **0.034** | 7.39 | 4.48 | 0.100 | 7.88 | 4.58 | 0.087 |
| Age | | -0.09 | 0.06 | 0.105 | -0.06 | 0.06 | 0.349 | 0.24 | 0.16 | **0.036** | 0.25 | 0.12 | **0.042** |
| Sex: female | | 1.45 | 1.08 | 0.182 | 1.63 | 1.10 | 0.141 | 4.42 | 2.02 | **0.030** | 3.62 | 2.08 | 0.084 |

^a^ Robust linear regression indicates that DBP was higher in dementia cases than controls (adjusted model 1: F = 4.97, df = 3, p = 0.0023)

^b^ DBP was higher in AD and VaD cases (adjusted model 2: F = 3.27, df = 5, p = 0.0072)

Total n = 226. Abbreviations: AD = Alzheimer’s disease; VaD = vascular dementia; DBP = diastolic BP; SBP = systolic BP; SE = standard error. Significant p-values are denoted in **bold**.

**Supplementary Table 3 Elevated pre-dementia BP in dementia and by disease subtype**

| Variable | | Pre-dementia DBP (mmHg) | | | | | | Pre-dementia SBP (mmHg) | | | | | |
| --- | --- | --- | --- | --- | --- | --- | --- | --- | --- | --- | --- | --- | --- |
|  | | Unadjusted analysis | | | Adjusted analysis | | | Unadjusted analysis | | | Adjusted analysis | | |
|  | | β | Rob-ust SE | *P*-value | β | Rob-ust SE | *P*-value | β | Rob-ust SE | *P*-value | β | Rob-ust SE | *P*-value |
| Model 1 (dementia vs controls)^a^ | | | | | | | | | | | | | |
| Dementia | | 6.37 | 1.50 | **<0.001** | 5.98 | 1.53 | **<0.001** | 8.14 | 2.97 | **0.007** | 6.72 | 2.88 | **0.021** |
| Age | | 0.02 | 0.09 | 0.784 | -0.01 | 0.08 | 0.913 | 0.48 | 0.17 | **0.004** | 0.40 | 0.17 | **0.017** |
| Sex: female | | 3.06 | 1.35 | **0.026** | 2.06 | 1.34 | 0.126 | 8.56 | 2.61 | **0.001** | 6.55 | 2.64 | **0.014** |
| Model 2 (dementia subtype vs controls)^b^ | | | | | | | | | | | | | |
| Cohort | AD | 4.79 | 2.42 | **0.050** | 4.72 | 2.36 | **0.047** | 4.90 | 4.45 | 0.273 | 6.05 | 4.27 | 0.159 |
|  | Mixed | 7.94 | 1.90 | **<0.001** | 7.41 | 2.06 | **<0.001** | 11.2 | 3.98 | **0.005** | 7.54 | 4.09 | 0.067 |
|  | VaD | 5.61 | 1.56 | **<0.001** | 5.04 | 1.99 | **0.013** | 7.21 | 2.09 | **0.001** | 5.90 | 2.80 | **0.037** |
| Age | | 0.02 | 0.09 | 0.784 | -0.03 | 0.08 | 0.685 | 0.48 | 0.17 | **0.004** | 0.39 | 0.16 | **0.020** |
| Sex: female | | 3.06 | 1.35 | **0.026** | 1.81 | 1.41 | 0.203 | 8.56 | 2.61 | **0.001** | 6.41 | 2.83 | **0.025** |

^a^ Robust linear regression indicates all-cause dementia was a significant predictor of both higher pre-dementia DBP and SBP compared to controls (adjusted model 1 DBP: *F* = 7.45, *df* = 3 and *p* = 0.0001; adjusted model 1 SBP: *F* = 9.48, *df* = 3 and *p* < 0.0001).

^b^ Robust linear regression also indicated that AD (*n* = 42), Mixed (*n* = 26), and VaD (*n* = 5) dementia subtypes were significant predictors of higher pre-dementia DBP compared to controls (adjusted model 2 DBP: *F* = 5.77, *df* = 5 and *p* < 0.0001) and that VaD was a significant predictor of higher pre-dementia SBP compared to controls (adjusted model 2 SBP: *F* = 6.53, *df* = 5 and *p* < 0.0001).

Total *n* = 137. Abbreviations: DBP = diastolic BP; SBP = systolic BP; SE = standard error. Significant *p*-values are denoted in **bold**.

**A**

**B**

**C**

**D**

**Supplementary Figure 1 Relationship between elevated late-life DBP and SBP and higher frontal and parietal arteriolosclerosis scores**. Individuals with elevated late-life DBP had significantly higher (**A**) frontal arteriolosclerosis scores in the combined mixed and vascular dementia groups (ordered logistic regression: adjusted OR per additional frontal arteriolosclerosis score point 1.07, 95% CI 1.03-1.11, *p* = 0.001), and elevated late-life DBP was also associated with higher (**B**) parietal arteriolosclerosis scores in the Alzheimer’s disease and combined mixed dementia and vascular dementia groups (adjusted OR per additional parietal arteriolosclerosis score point 1.05, 95% CI 1.01-1.09, *p* = 0.007). A similar relationship was observed for individuals with elevated late-life SBP: this was associated with higher (**C**) frontal arteriolosclerosis scores in vascular and mixed dementia (adjusted OR per additional frontal arteriolosclerosis score point 1.03, 95% CI 1.01-1.05, *p* = 0.001), and (**D**) higher parietal arteriolosclerosis scores in Alzheimer’s disease, mixed dementia, and vascular dementia (adjusted OR per additional parietal arteriolosclerosis score point 1.02, 95% CI 1.01-1.04, *p* = 0.017).

Abbreviations: AD = Alzheimer’s disease; VaD = vascular dementia; DBP = diastolic blood pressure; SBP = systolic blood pressure.

**Supplementary Table 4 Donors with elevated late-life SBP had higher frontal and parietal arteriosclerosis scores**

| Variables | | | Unadjusted analysis | | | Adjusted analysis | | |
| --- | --- | --- | --- | --- | --- | --- | --- | --- |
|  |  |  | Odds ratio | 95% CI | *P-*value | Odds ratio | 95% CI | *P-*value |
| Frontal arteriosclerosis score | | | | | | | | |
| Cohort | | AD | 1.25 | 0.73-2.16 | 0.414 | 1.49 | 0.81-2.72 | 0.199 |
|  | | Mixed | 6.36 | 2.99-13.5 | **<0.001** | 5.29 | 2.46-11.4 | **<0.001** |
|  | | VaD | 19.4 | 7.74-48.5 | **<0.001** | 19.3 | 7.45-49.9 | **<0.001** |
| Age (y) | | | 1.04 | 1.01-1.07 | **0.002** | 1.02 | 0.99-1.06 | 0.143 |
| SBP (65+ y) | | | 1.03 | 1.02-1.05 | **<0.001** | 1.03 | 1.01-1.05 | **0.001** |
| Parietal arteriosclerosis score | | | | | | | | |
| Cohort | AD | | 1.91 | 1.07-3.44 | **0.030** | 2.49 | 1.29-4.77 | **0.006** |
|  | Mixed | | 9.01 | 4.38-18.5 | **<0.001** | 8.21 | 3.98-16.9 | **<0.001** |
|  | VaD | | 18.46 | 5.90-57.8 | **<0.001** | 19.6 | 5.57-68.7 | **<0.001** |
| Age (y) | | | 1.03 | 1.01-1.06 | **0.005** | 1.04 | 1.01-1.07 | **0.023** |
| SBP (65+ y) | | | 1.03 | 1.01-1.05 | **0.001** | 1.02 | 1.00-1.04 | **0.017** |

The effect of higher SBP on arteriosclerosis score was significant in an ordered logistic regression model, adjusting for the effects of age and dementia subtype (adjusted OR per additional frontal arteriosclerosis score point: 1.03, 95% CI 1.01-1.05, *p* = 0.001; parietal arteriosclerosis score point: 1.02, 95% CI 1.01-1.04, *p* = 0.017). Significant *p*-values are denoted in **bold**.

Frontal *n* = 226, parietal *n* = 204

Abbreviations: AD = Alzheimer’s disease; VaD = vascular dementia*;* CI = confidence interval; SBP = systolic BP.

**Supplementary Table 5 Relationship between frontal and parietal CAA scores and late-life SBP**

|  | | | Unadjusted analysis | | | Adjusted analysis | | |
| --- | --- | --- | --- | --- | --- | --- | --- | --- |
|  |  |  | Odds ratio | 95% CI | *P-*value | Odds ratio | 95% CI | *P-*value |
| Frontal CAA score | | | | | | | | |
| Cohort | | AD | 5.72 | 3.23-10.14 | **<0.001** | 4.61 | 2.56-8.30 | **<0.001** |
|  | | Mixed | 3.81 | 1.56-9.30 | **0.003** | 3.73 | 1.55-9.00 | **0.003** |
|  | | VaD | 0.55 | 0.16-1.87 | 0.334 | 0.46 | 0.14-1.53 | 0.207 |
| Age (y) | | | 0.97 | 0.95-0.99 | **0.008** | 0.99 | 0.96-1.02 | 0.497 |
| SBP (65+ y) | | | 1.01 | 1.00-1.03 | 0.109 | 1.02 | 1.00-1.03 | **0.038** |
| Parietal CAA score | | | | | | | | |
| Cohort | AD | | 3.57 | 2.06-6.19 | **<0.001** | 4.37 | 2.43-7.85 | **<0.001** |
|  | Mixed | | 2.67 | 1.12-6.39 | **0.027** | 2.58 | 1.04-6.39 | **0.040** |
|  | VaD | | 0.54 | 0.16-1.76 | 0.303 | 0.52 | 0.16-1.64 | 0.263 |
| Age (y) | | | 1.00 | 0.98-1.03 | 0.784 | 1.02 | 0.99-1.06 | 0.169 |
| SBP (65+ y) | | | 1.01 | 0.99-1.02 | 0.420 | 1.01 | 0.99-1.02 | 0.314 |

The effect of higher SBP on frontal CAA score was significant in an ordered logistic regression model, adjusting for the effects of age and dementia subtype (adjusted OR per additional frontal CAA score point: 1.02, 95% CI 1.00-1.03, *p* = 0.038). The effect of higher SBP was not significant with respect to parietal CAA score. Significant *p*-values are denoted in **bold**.

Frontal *n* = 226, parietal *n* = 218

Abbreviations: AD = Alzheimer’s disease; VaD = vascular dementia*;* CI = confidence interval; SBP = systolic BP; CAA = cerebral amyloid angiopathy.

**
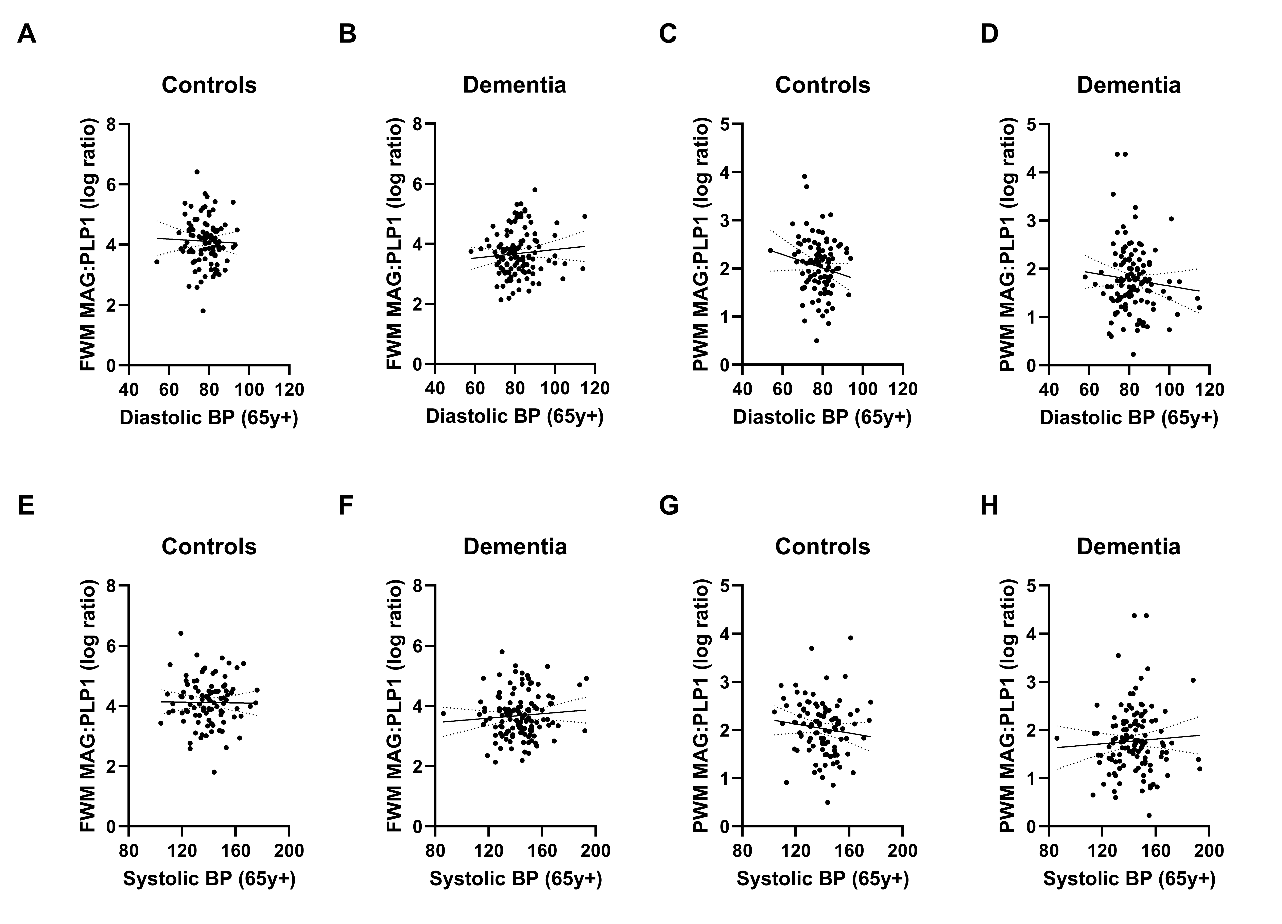
**

**Supplementary Figure 2 Relationship between SBP and DBP and MAG:PLP1 ratios was unchanged in the white matter**. Late-life DBP was not associated with (**A**) FWM MAG:PLP1 ratios in controls (Pearson’s correlation: ns, *n* = 99), or with (**B**) FWM MAG:PLP1 in dementia cases (ns, *n* = 125). Similarly, late-life DBP was not associated with (**C**) PWM MAG:PLP1 in controls (ns, *n* = 99), or with (**D**) PWM MAG:PLP1 ratios in dementia cases (ns, *n* = 125). Late-life SBP did not correlate with white matter MAG:PLP1 in dementia cases or controls in either brain region (**E-H**; ns). Each point represents a single case. The continuous and interrupted lines indicate the best-fit linear regression and 95% confidence intervals. Abbreviations: MAG:PLP1 = myelin-associated glycoprotein-proteolipid protein-1 ratio; FWM = frontal white matter; PWM = parietal white matter; y= years; BP = blood pressure.


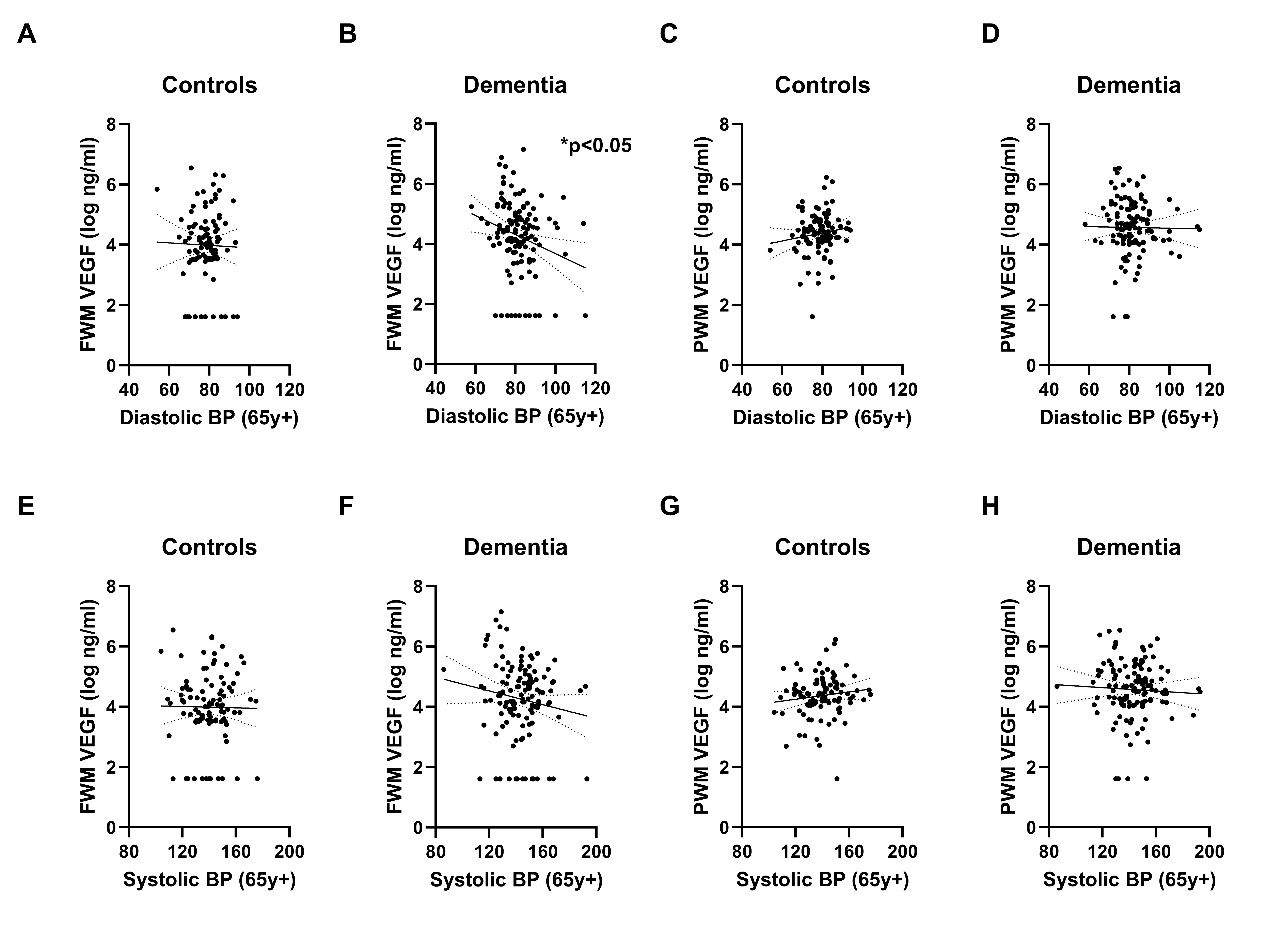


**Supplementary Figure 3 Elevated diastolic blood pressure in late life was associated with lower VEGF levels in the frontal white matter**. No relationship was seen between DBP and (**A**) FWM VEGF in controls (Pearson’s correlation: ns, *n* = 99), but late-life DBP was associated with lower (**B**) FWM VEGF in dementia cases (Pearson *r* = -0.2267, *p* = 0.0107, *n* = 126). DBP was unchanged in relation to both (**C**) PWM VEGF in controls (ns, *n* = 99), and to (**D**) PWM VEGF in dementia cases (ns, *n* = 125). Late-life SBP did not correlate with VEGF in dementia cases or controls in either brain region (**E-H**; ns). Each point represents a single case. The continuous and interrupted lines indicate the best-fit linear regression and 95% confidence intervals. Abbreviations: VEGF = vascular endothelial growth factor; FWM = frontal white matter; PWM = parietal white matter; y= years; BP = blood pressure.


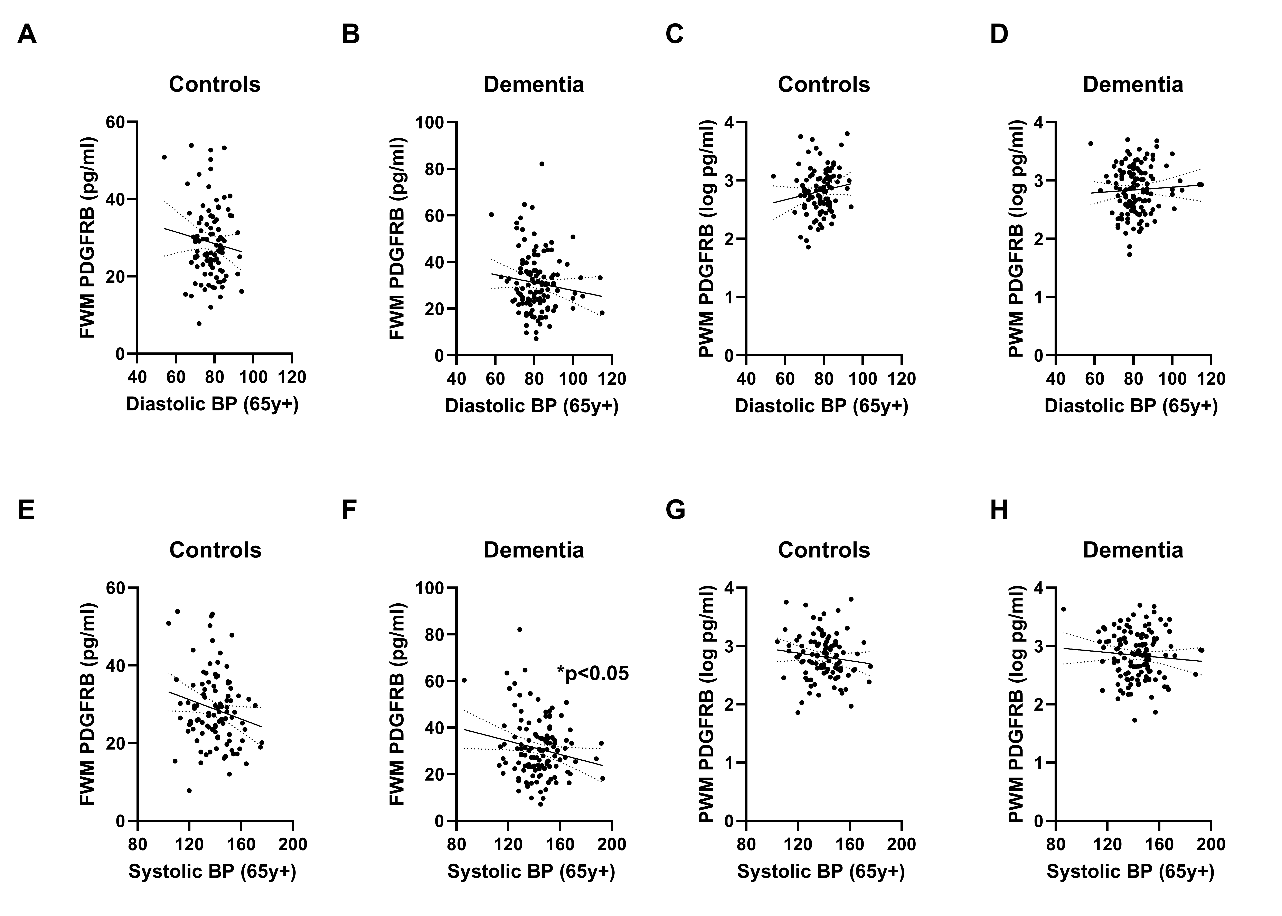


**Supplementary Figure 4 Elevated diastolic blood pressure in late life was associated with lower PDGFRB levels in the frontal white matter**. Late-life DBP did not correlate with FWM PDGFRB in (**A**) controls or (**B**) dementia, or with PWM PDGFRB (**C**) in controls or (**D**) dementia. Late-life SBP did not correlate with FWM PDGFRB in (**E**) controls, but late-life SBP was associated with lower (**F**) FWM PDGFRB in dementia cases (Pearson’s correlation: *r* = -0.1821, *p* = 0.0413, *n* = 126). There were no significant correlations between late-life SBP and PWM PDGFRB in either (**G**) controls and (**H**) dementia (both ns). Each point represents a single case. The continuous and interrupted lines indicate the best-fit linear regression and 95% confidence intervals. Abbreviations: PDGFRB = platelet-derived growth factor receptor β; FWM = frontal white matter; PWM = parietal white matter; y= years; BP = blood pressure.
